# The Apparent Diffusion Coefficient of Diffusion-Weighted Whole-Body Magnetic Resonance Imaging Affects the Survival of Multiple Myeloma Independently

**DOI:** 10.3389/fonc.2022.780078

**Published:** 2022-03-04

**Authors:** Bei Zhang, Bingyang Bian, Yanjiao Zhang, Li Zhang, Rongkui Zhang, Jiping Wang

**Affiliations:** ^1^Department of Radiology, First Hospital of Jilin University, Changchun, China; ^2^Department of Hematology, First Hospital of Jilin University, Changchun, China

**Keywords:** DW-MRI, multiple myeloma, apparent diffusion coefficient, survival, prognosis

## Abstract

**Background:**

Diffusion-weighted whole-body MRI (DW-MRI) is increasingly used to evaluate bone diseases of multiple myeloma (MM), but there is lack of quantitative indicator for DW-MRI to reflect the prognosis of MM. Apparent diffusion coefficient (ADC) values in DW-MRI has potential correlations between some indexes of MM, but the influence of ADC on MM survival needs to be further verified.

**Methods:**

A total of 381 newly diagnosed MM patients were enrolled in the study to analyze the effect of ADC values in DW-MRI on progression-free survival (PFS) and overall survival (OS). The Kaplan–Meier method was used to perform univariate survival analysis, and the Cox proportional hazards model was used for multivariate analysis. In addition to the ADC value, genetic and serological indexes were also included.

**Results:**

The survivals were observed in univariate ADC stratification with median PFS of 52.0, 45.0, 34.0, and 26.0 months (the unit of ADC value was 10^−3^ mm^2^/s; the ADC ranges were ADC < 0.4886, 0.4886 ≤ ADC < 0.6545, 0.6545 ≤ ADC < 0.7750, and ADC ≥ 0.7750; 95% CI, 43.759–62.241, 46.336–53.664, 39.753–46.247, and 27.812–32.188). The OS were 81.0, 61.0, 47.0, and 36.0 months (*p* < 0.001; 95% CI, 71.356–82.644, 67.630–70.370, 57.031–60.969, and 36.107–43.893). In Cox proportional hazards model, the ADC value was considered to be an independent risk factor affecting PFS and OS of MM (both *p* < 0.001).

**Conclusions:**

This study supports that ADC in DW-MRI may independently stratify MM patients and better predict their prognosis. The combined use of DW-MRI and other parameters allows more accurate evaluation of MM survival.

**Trial Registration:**

http://www.chictr.org.cn/showproj.aspx?proj=49012, ChiCTR2000029587.

## Introduction

Multiple myeloma (MM) is a malignant hematological disease caused by abnormal clonal proliferation of plasma cells (1). MM ranks second in hematological malignancies, which is considered to be incurable at present (2). About 80%–90% of MM patients will exhibit different degrees of MM-related bone diseases (3). The severity of bone disease increases mortality (4). The incidence rate of MM worldwide is increasing, and more than 140,000 people are diagnosed as MM every year (5, 6). The overall survival (OS) of MM patients was 30 months. Thalidomide treatment could prolong the OS to 46 months and bone marrow transplantation to 48 months (7). Although with the change of treatment methods the 5-year survival rate of MM has increased from 28% to 56% (1975 to 2012) (5), the imaging methods that can effectively predict the prognosis of the disease still need to be explored. Therefore, a reliable, noninvasive imaging method for survival prediction is urgently needed. The diffusion-weighted whole-body MRI (DW-MRI) has recently been developed and has evolved as a comprehensive and standardized diagnostic algorithm for MM lesions. Plumbing reliable imaging parameters to quantify these survival conditions is a new challenge to medical imaging.

The International Myeloma Working Group (IMWG) recommends DW-MRI as an effective imaging method to detect MM bone disease (8). DW-MRI has been applied to MM because of its relatively excellent sensitivity compared with FDG PET/CT and whole-body low-dose CT (9). DW-MRI has been proven to be an effective tool not only for MM diagnosis but also for outcome prediction after chemotherapy, exerting an increasing influence on the treatment management of MM (10). Even though DW-MRI is now gradually replacing traditional imaging methods of MM diagnosis (11), its implementation is not complete in predicting survival due to lack of relevant studies. Therefore, through high sensitivity, the survival time in MM can be detected in the DW-MRI images, which holds promise for the prediction of survival condition associated with personalized treatment. However, whether the imaging information about DW-MRI can predict the prognosis of MM patients and provide hints of disease management remains to be discussed.

Some studies have found that the change of ADC value could reflect the response to disease treatment before the size and number of MM bone lesion (12, 13). MM patients with nonequivalent bone lesions on DW-MRI mean worse prognosis than those negative ones (14). In addition to the treatment response, ADC values vary with disease progression (15). ADC seems to confer a quantitative method of patients with MM, including biochemical index and treatment response. The accurate and reliable correlation between ADC and MM survival, therefore, still represents an unmet need to adequately tailor therapeutic management. Taking into consideration relative convince and maturity in application, the ADC of DW-MRI becomes the potential parameter for prognostic prediction. The relationship between ADC value and the survival time of MM patient remains inconclusive yet. Whether the prognosis and survival status of MM patients can be presented by ADC needs to be further demonstrated.

The aim of our study was to evaluate whether ADC value measured in newly diagnosed MM patients can be a meaningful factor of disease survival.

## Methods

### Patients

This was a retrospective study, which has been approved by the ethics committee of the First Hospital of Jilin University. Patients with newly diagnosed MM were enrolled from January 2010 to June 2021.

The inclusion criteria were as follows: (1) All of the MM patients meet the diagnostic criteria of the International Myeloma Working Group (IMWG); (2) All patients were newly diagnosed MM at the time of inclusion; (3) whole-body diffusion-weighted imaging (WB-DWI) was performed before treatment; (4) The interval between initial chemotherapy and WB-DWI was within 1 week; (5) All the patients enrolled were symptomatic or active MM with detectable M protein in blood or urine; (6) All the patients received at least four courses of first-line treatment based on bortezomib, and the treatment response was evaluated; (7) All patients received regular (every 3 months) inpatient services, outpatient services, and telephone follow-up.

The exclusion criteria are as follows: (1) Extramedullary plasmacytoma was found when MM was newly diagnosed and (2) Patients with bone diseases other than MM affecting ADC value measurement.

### Treatment

All enrolled patients received at least three to four courses of bortezomib-based induction chemotherapy, including bortezomib and dexamethasone (VD); bortezomib, cyclophosphamide, and dexamethasone (VCD); and bortezomib, thalidomide, and dexamethasone (VTD). Treatment response was assessed by the National Comprehensive Cancer Network guidelines on MM. Patients eligible for autologous stem cell transplantation underwent ASCT after induction chemotherapy. Bortezomib-based maintenance therapy was performed after ASCT for at least 2 years or longer.

### Follow-Up

The treatment response evaluation referred to the standards of IMWG (16). Progression-free survival (PFS) was calculated from the date of the enrollment until progression or death for any cause. Overall survival (OS) was calculated from the date of enrollment until death for any cause or the last follow-up.

### DW-MRI Examination

WB-DWIs were performed on a 3.0-T Ingenia Elition MR imaging scanner (Philips, The Netherlands) with a rolling table platform, integrated coil, two surface coils, head coil, and neck coil. DWI, T1 Dixon, as well as T2 STIR were included in the scanning protocol (**Table 1**). The scanning position was supine with head first. The whole image scanning was divided into six sections (head, chest, abdomen, pelvis, thigh, and shank). The whole spine scan was divided into three sections (cervical spine, thoracic spine, and lumbosacral spine). The whole scanning time was about 45 min.

**Table 1:** WB-DWI scanning parameters.

	DWI coronal	DWI axial	T2 STIR coronal	T2 FSE sagittal	T1 Dixon coronal	T1 FSE sagittal
Number of slices	48	36	24	12	24	12
FOV	400	400	400	400	400	400
Thickness (mm)	1.5	3	3	3	3	3
TR (ms)	5200	5200	8600	2320	480	420
TE (ms)	110	80	50	50	40	50
TI (ms)	180	160	150	160		
Image matrix	128 × 128	256 × 256	256 × 256	128 × 128	256 × 256	256 × 192
Number of excitations	4	2	2	2	2	2
*b*-values (s/mm^2^)	0/1,000	0/1,000				
Acquisition time (min)	4	2	2	2	1	2

FOV, field of view; STIR, short-time inversion recovery; TE, echo time; TI, inversion time; TR, repetition time.

### DW-MRI Data Processing

The images were processed by EWS workstation (Philips, The Netherlands). The whole body was divided into nine bone regions, including skull, bilateral ribs, cervical spine, thoracic spine, lumbar spine, pelvis, bilateral femur, bilateral tibiofibula, and bilateral humerus. Identification criteria of positive bone areas are as follows: (1) If a bone region has one or more abnormal signals ≥5 mm, it is considered that the bone region has MM lesions and was marked as positive and (2) If diffuse lesions are found in one bone, the bone will be regarded as a lesion, then this bone region was recorded as positive.

The criteria for the selection of lesions for ROI placement include the following: (1) Select a unique ROI for each bone region; 2) For MM patients with multiple focal pattern, the largest lesion in each region was selected as the target for ROI placement; (3) The minimum size of lesion selected for measurement was 5 mm in diameter; (4) For patients with diffuse pattern, the bone in each region with relatively large cross-sectional area and uniform signal was selected to place the ROI; (5) The ROI area for calculating ADC value is 10 mm^2^ fixedly; and (6) The ROI was selected in the center of the lesion on the maximum slice as far as possible, avoiding measurement of the edge to avoid heterogeneous signals.

The steps for ADC value calculation are as follows: (1) The average ADC value of all the bone regions was viewed as the final ADC value of a patient and (2) The ADC value of each patient was measured separately by two radiologists who were blind to clinical and biological data, and the average value of the two was the ADC value of each patient. Intraclass correlation coefficient (ICC) values of the two observers (0.84, 95% CI: 0.87∼0.90) indicated strong reliability; and (3) Both radiologists measured the ROI of the enrolled patients twice, and within-session reliability was excellent with interclass correlation coefficient of 0.90 and 0.87.

In the evaluation of diagnostic accuracy of different sequences of DW-MRI, the gold standard was identified as pathological examination or combined clinical/imaging follow-up data.

In the Kaplan–Meier method, the grouping factors of ADC values refer to the results of optimal stratification. The groups were divided into the following three groups: ADC < 0.4886, 0.4886 ≤ ADC < 0.6545, 0.6545 ≤ ADC < 0.7750, and ADC ≥ 0.7750 (The unit of ADC value is 10^−3^ mm^2^/s).

### Clinical Data Acquisition

In addition to imaging data, other baseline clinical parameters including age, gender, RISS stage, treatment schedule, FISH results, serum β2-MG, LDH, and albumin were obtained from the hospital information system (Shanghai Union Networks and Information, China).

### Statistical Analysis

Data were analyzed using SPSS 25.0 (International Business Machines Corporation, USA). Continuous variables were expressed as mean ± standard deviation (SD) or median (interquartile range) according to whether they were normal distribution. Categorical variables were expressed as frequency (percentage).

Optimal stratification, a statistical method based on log-rank statistics, was used to solve specific threshold values with the continuous covariate (ADC value of MM lesion). The most significant *p*-value was found by means of the log-rank *χ*^2^ statistic to derive the sex-specific cutoffs at which patients were best separated with respect to time for mortality. It is appropriate to identify survival-related thresholds using optimal stratification, which was previously described in the literature (17). The cutoffs obtained by this method were then used to classify the patients’ ADC values. The Kaplan–Meier method was used to generate survival curves, and the log-rank test was used to compare the differences in patient survival. The results of multivariate analysis using the Cox proportional hazards model are shown as the hazard ratios (HRs) and 95% confidence intervals (CIs). The results of all tests were bilateral. *p* < 0.05 was considered statistically significant.

## Results

### Baseline Characteristics

A total of 381 patients with MM were enrolled in the study. Their mean age was 60.61 ± 9.40 years, and MM affected more men than women. The patients’ clinical characteristics including Revised International Staging System (RISS) stage, immunotype, and treatment response are shown in **Table 2**.

**Table 2:** Baseline demographics and clinical characteristics of the patient population.

Parameters	Baseline demographics and clinical characteristics
No. of subjects	381
Sex (M/F)	225/156 (59.1%/40.9%)
Age (mean) (year)	60.61 ± 9.40
RISS Stage	
Stage I	38 (10.0%)
Stage II	236 (61.9%)
Stage III	107 (28.1%)
Type	
IgA-λ	31 (8.1%)
IgD-λ	35 (9.2%)
IgG-	123 (32.3%)
λ	68 (17.8%)
IgA-κ	37 (9.7%)
IgG-κ	58 (15.2%)
κ	29 (7.6%)
Therapeutic response	
CR	131 (34.4%)
sCR	85 (22.3%)
PR	74 (19.4%)
VGPR	51 (13.4%)
SD	23 (6.0%)
PD	17 (4.5%)

CR, complete response; PD, progressive disease; PR, partial response; RISS, Revised International Staging System; sCR, strict complete response; SD, stable disease; VGPR, very good partial response.

### Diagnostic Results of Different Sequences in DW-MRI

Each MM patient had nine bone regions, and a total of 381 patients were enrolled. A total of 3,429 bone regions were included in the study. WB-DWI combined with T2 STIR showed better accuracy in diagnosing MM than WB-DWI and T2 STIR alone (**Tables 3** and **4**).

**Table 3:** Number of diagnostic results of all the bone regions for WB-DWI, WB-STIR, and combined WB-DWI + WB-STIR.

Imaging modalities	True positive	True negative	False positive	False negative
WB-DWI	2,105	1,069	12	243
WB-STIR	1,955	809	272	393
WB-DWI + WB-STIR	2,239	1,035	46	109

**Table 4:** Comparison of diagnostic results among WB-DWI, WB-STIR, and combined WB-DWI + WB-STIR.

Parameters	WB-DWI	WB-STIR	WB-DWI + WB-STIR	*p*
Overall accuracy	92.6%	80.6%	95.5%	<0.001^*^
Sensitivity	89.7%	83.3%	95.4%	<0.001^*^
Specificity	98.9%	74.8%	95.7%	<0.001^*^
PPV	99.4%	87.8%	98.0%	<0.001^*^
NPV	81.5%	67.3%	90.5%	<0.001^*^

^*^Significant difference.

PPV, positive predictive value; NPV, negative predictive value.

### Univariate Analysis of PFS in MM Patients

The median PFS time was 38.0 months (95% CI: 35.182–40.818), and the estimated 3- and 5-year PFS rates were 44.9% and 9.7%, respectively.

**Figure 1:**
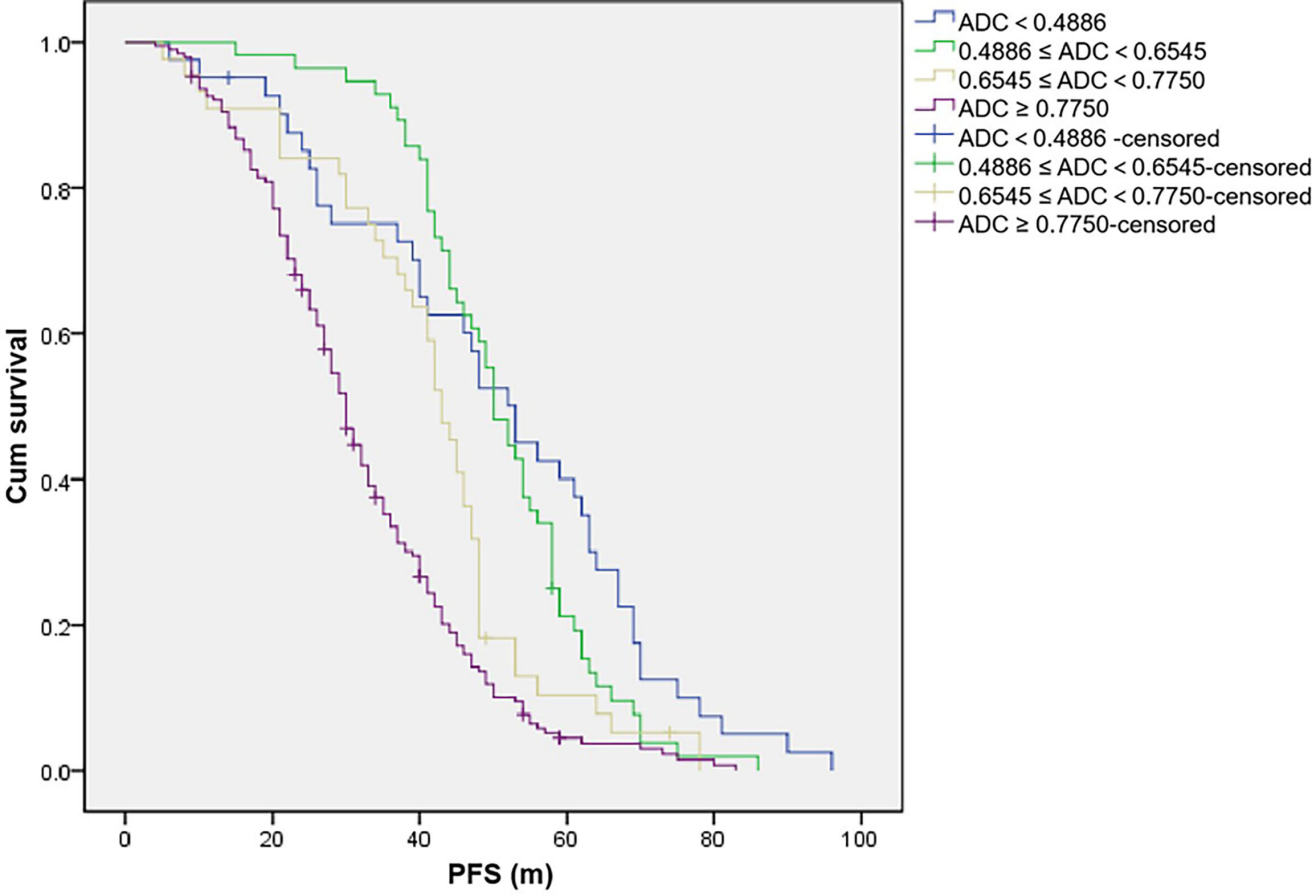
The unit of apparent diffusion coefficient (ADC) value is 10^−3^ mm^2^/s. Progression-free survival (PFS) of patients with different ADC values.

Univariate analysis (**Table 5**; **Figure 1**) showed that different grouping levels of ADC values of WB-DWI at baseline may indicate different PFS events (*p* < 0.001), with 3-year PFS rates (ADC < 0.4886, 0.4886 ≤ ADC < 0.6545, 0.6545 ≤ ADC < 0.7750, and ADC ≥ 0.7750, the unit of ADC value was 10^−3^ mm^2^/s) of 65.2%, 82.3%, 60.8%, and 26.6%, respectively. The ≥65-year-old group also indicated poor PFS (*p* = 0.006), and the 3-year PFS rates were 40.0% and 47.7%, respectively. Similar results were found in the group with or without p53 gene deletion (*p* = 0.042), and the 3-year PFS rates were 42.7% and 45.4%, respectively. In addition, IGH rearrangement may also mean poor PFS (<0.001) with 3-year PFS rates of 29.9% and 48.1%. However, no difference was detected in PFS between male and female patients (*p* = 0.163), with a 3-year PFS rate of 46.2% and 42.9% (**Table 5**), respectively. The serum albumin and β2-MG levels significantly affected the PFS of the patients (*p* < 0.001). However, the serum LDH level, RB1 deletion, and 1q21 amplification did not have a statistically significant effect on PFS.

**Table 5:** Univariate analysis of PFS in MM patients.

Factors	Number of cases	Median PFS (m)	95% CI	*p*-value
Age (year)				
<65	241	41	38.170–43.830	0.006
≥65	140	35	28.418–41.582	
Gender				
Male	225	39	35.989–42.011	0.163
Female	156	37	31.419–42.581	
Albumin (g/L)				
<35	149	37	32.588–41.412	0.004
≥35	232	44	40.498–47.502	
β2-MG (mg/ml)				
<3.5	174	46	42.116–49.884	0.001
3.5 ≤ β2-MG < 5.5	86	43	38.796–47.204	
≥5.5	121	35	30.211–39.789	
LDH (U/L)				
<245	142	43	39.976–46.024	0.105
≥245	239	40	36.808–43.192	
P53				
Deletion	75	42	34.773–49.227	0.042
Nondeletion	306	41	38.809–43.191	
RB1				
Deletion	61	40	32.119–47.881	0.167
Nondeletion	320	41	38.743–43.257	
1q21				
Amplification	58	40	34.995–45.005	0.536
Nonamplification	323	41	38.813–43.187	
IGH				
Rearrangement	67	30	12.078–47.922	<0.001
Nonrearrangement	314	42	39.948–44.052	
ADC value (×10^−3^ mm^2^/s)				
ADC < 0.4	22	48	39.468–56.532	<0.001
0.4 ≤ ADC < 0.6	60	53	48.202–57.798	
0.6 ≤ ADC < 0.8	92	43	39.518–46.482	
0.8 ≤ ADC < 1.0	99	33	28.542–37.458	
1.0 ≤ ADC < 1.2	74	29	25.607–32.393	
ADC ≥ 1.2	34	21	17.757-24.243	

### Univariate Analysis of OS in MM Patients

The median OS time was 51.0 months (95% CI: 47.160–54.840) with 94.0 months of median follow-up. The estimated 5-year OS rates were 32.5%.

**Figure 2:**
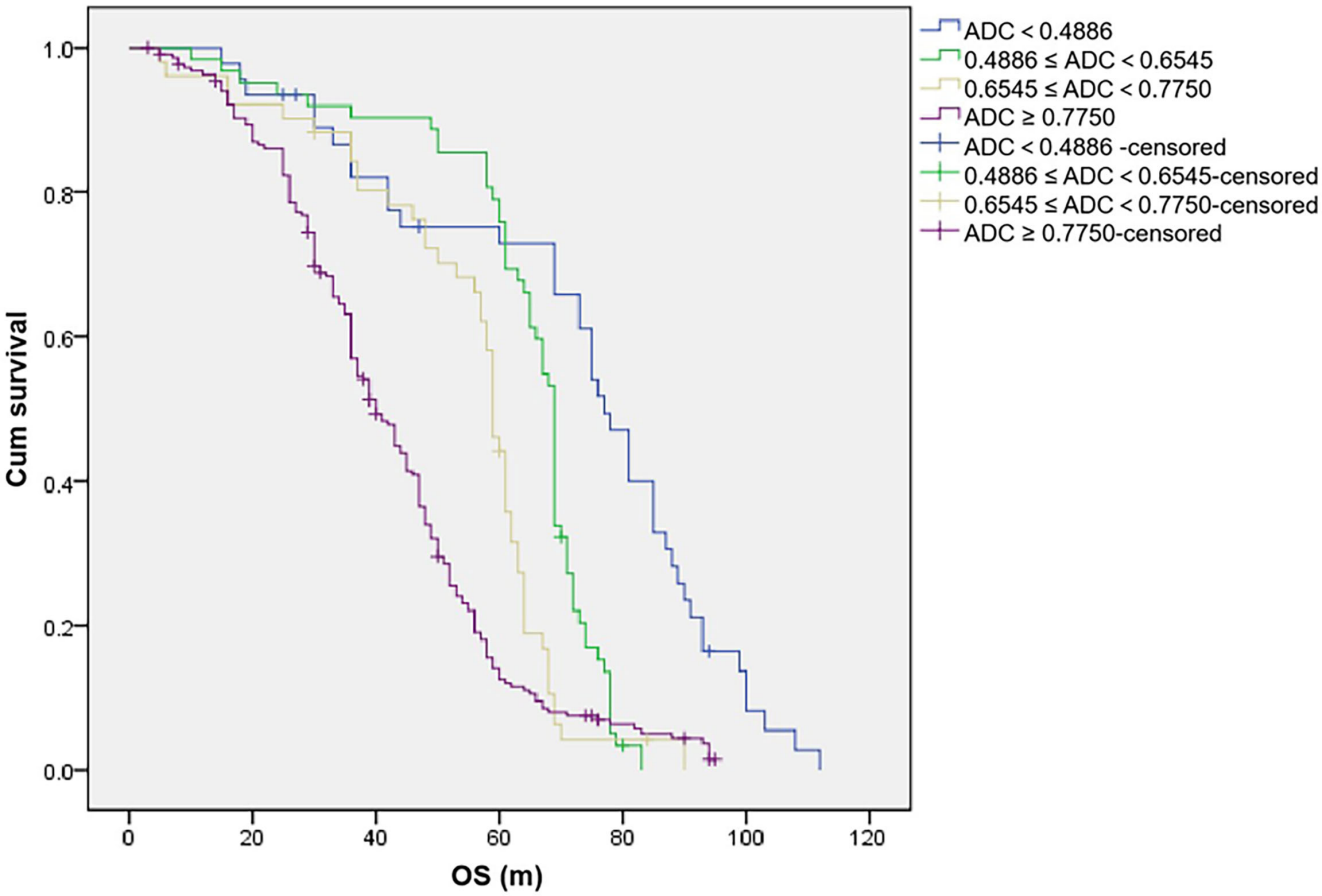
The unit of apparent diffusion coefficient (ADC) value is 10^−3^ mm^2^/s. Overall survival (OS) of patients with different ADC values.

Significant difference was found in OS according to different ADC values in WB-DWI (*p* < 0.001), with 5-year OS rates (ADC < 0.4886, 0.4886 ≤ ADC < 0.6545, 0.6545 ≤ ADC<0.7750, and ADC ≥ 0.7750, the unit of ADC value was 10^−3^ mm^2^/s) of 67.4%, 75.8%, 41.2%, and 11.3%, respectively (**Table 6**; **Figure 2**). Compared with patients without genetic variation, patients with IGH rearrangement had shorter median OS (median OS, 36.0 months vs 52.0 months, *P* < 0.001; 5-year survival rate, 28.4% vs 33.4%). The serum β2-MG, albumin, and LDH level significantly affected the OS of the patients (*p* < 0.005). The ≥65- and<65-year-old group did not indicate poorer OS (*p* = 0.251), and the 5-year OS rates were 35.0% and 31.1%, respectively. However, 1q21 amplification, P53, RB1 deletion, and gender did not have a statistically significant effect on OS.

**Table 6:** Univariate analysis of OS in MM patients.

Factors	Number of cases	Median OS (m)	95% CI	*p*-value
Age (year)				
<65	241	52	47.377–56.623	0.251
≥65	140	48	39.113–56.887	
Gender				
Male	225	54	49.730–58.270	0.163
Female	156	48	44.356–51.644	
Albumin (g/L)				
<35	149	45	38.732–51.268	0.001
≥35	232	58	54.116–63.884	
β2-MG (mg/ml)				
<3.5	174	55	47.483–62.517	0.036
3.5 ≤ β2-MG < 5.5	86	59	54.116–63.884	
≥5.5	121	47	42.144–51.856	
LDH (U/L)				
<245	142	56	49.881–62.119	0.047
≥245	239	49	44.266–53.734	
P53				
Deletion	75	50	47.359–56.641	0.171
Nondeletion	306	52	42.676–57.324	
RB1				
Deletion	61	49	39.578–58.422	0.346
Nondeletion	320	52	47.438–56.562	
1q21				
Amplification	58	50	42.053–57.947	0.672
Nonamplification	323	51	46.528–55.472	
IGH				
Rearrangement	67	36	18.337–53.663	<0.001
Nonrearrangement	314	52	47.974–56.026	
ADC value (×10^−3^ mm^2^/s)				
ADC < 0.4	17	81	63.187–98.813	<0.001
0.4 ≤ ADC < 0.6	56	71	68.590–73.410	
0.6 ≤ ADC < 0.8	89	59	57.136–60.864	
0.8 ≤ ADC < 1.0	99	47	44.075–49.925	
1.0 ≤ ADC < 1.2	82	37	28.772–45.228	
ADC ≥ 1.2	38	33	28.178–37.822	

### Multivariate Analysis of PFS in MM Patients

The factors that entered the Cox proportional hazards model analysis included ADC value, gene variations, serum album, β2-MG, LDH, and age. The results suggested that PFS was independently affected by ADC value, age, IGH rearrangement, and serum LDH (all *p* < 0.05, **Table 7**).

**Table 7:** Multivariate analysis of PFS in MM patients.

Parameters	B	SE	Wald	*df*	Sig.	Exp (B)	95% CI for Exp (B)	
							Lower	Upper
ADC value	2.040	0.238	73.681	1	<0.001	7.691	4.827	12.255
Age	0.017	0.006	7.867	1	0.005	1.017	1.005	1.029
Album	−0.004	0.008	0.185	1	0.667	0.996	0.981	1.013
β2-MG	0.001	0.003	0.035	1	0.852	1.001	0.994	1.007
LDH	−0.002	0.001	9.313	1	0.002	0.998	0.996	0.999
IGH rearrangement	0.378	0.166	5.195	1	0.023	1.459	1.054	2.019
P53 deletion	0.182	0.145	1.583	1	0.208	1.200	0.903	1.593

ADC, apparent diffusion coefficient.

### Multivariate Analysis of OS in MM Patients

Among the available prognostic parameters (ADC value, gene variations, serum album, β2-MG, LDH, and age), multivariate analysis showed that ADC value, age, and IGH rearrangement were independent prognostic factors of patients with OS (all *p* < 0.05, **Table 8**).

**Table 8:** Multivariate analysis of OS in MM patients.

Parameters	B	SE	Wald	*df*	Sig.	Exp (B)	95% CI for Exp (B)	
							Lower	Upper
ADC value	1.916	0.212	82.045	1	<0.001	6.795	4.489	10.286
Age	0.011	0.005	4.454	1	0.035	1.011	1.001	1.022
Album	−0.002	0.007	0.055	1	0.814	0.998	0.984	1.012
β2-MG	<0.001	0.003	<0.001	1	0.989	1.000	0.994	1.007
LDH	−0.001	0.001	3.118	1	0.077	0.999	0.998	1.000
IGH rearrangement	0.558	0.155	12.958	1	<0.001	1.747	1.289	2.368
P53 deletion	0.225	0.137	2.707	1	0.100	1.253	0.958	1.638

ADC, apparent diffusion coefficient; LDH, lactic dehydrogenase.

## Discussion

This study focused on the effects of ADC values together with other clinical parameters on PFS and OS in multiple myeloma. Based on the univariate and multivariate methods of survival analysis, it was found that the ADC value of DW-MRI at baseline was an independent risk factor affecting the prognosis of multiple myeloma. Given that this is a retrospective study, the potential influence of DW-MRI in MM disease management needs to be confirmed in many aspects.

**Figure 3:**
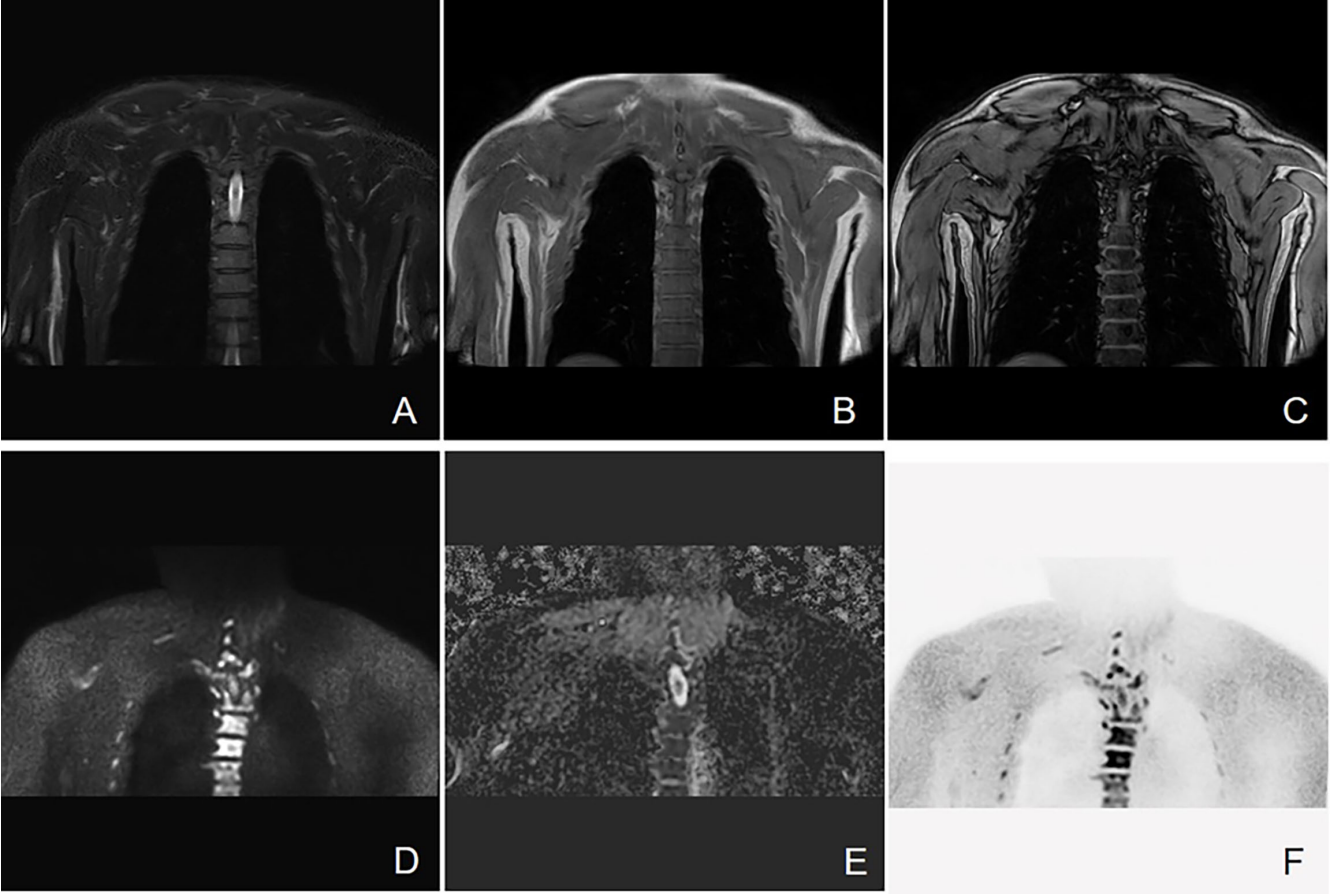
Active progressive MM disease. A case of a 63-year-old man who was diagnosed with MM (kappa light chain, RISS Stage III) is presented here. **(A–C)** Coronal images of T2 STIR, in-phase and out-phase of the chest. **(D–F)** Coronal images of DWI, ADC map, and inverted images, respectively. Diffuse abnormal signal is seen in the thoracic vertebrae, ribs, and right scapula within the scanning range, which show slightly low signal on T1WI and slightly high signal on T2 STIR. Significant high signal can be observed in DWI sequence.

**Figure 4:**
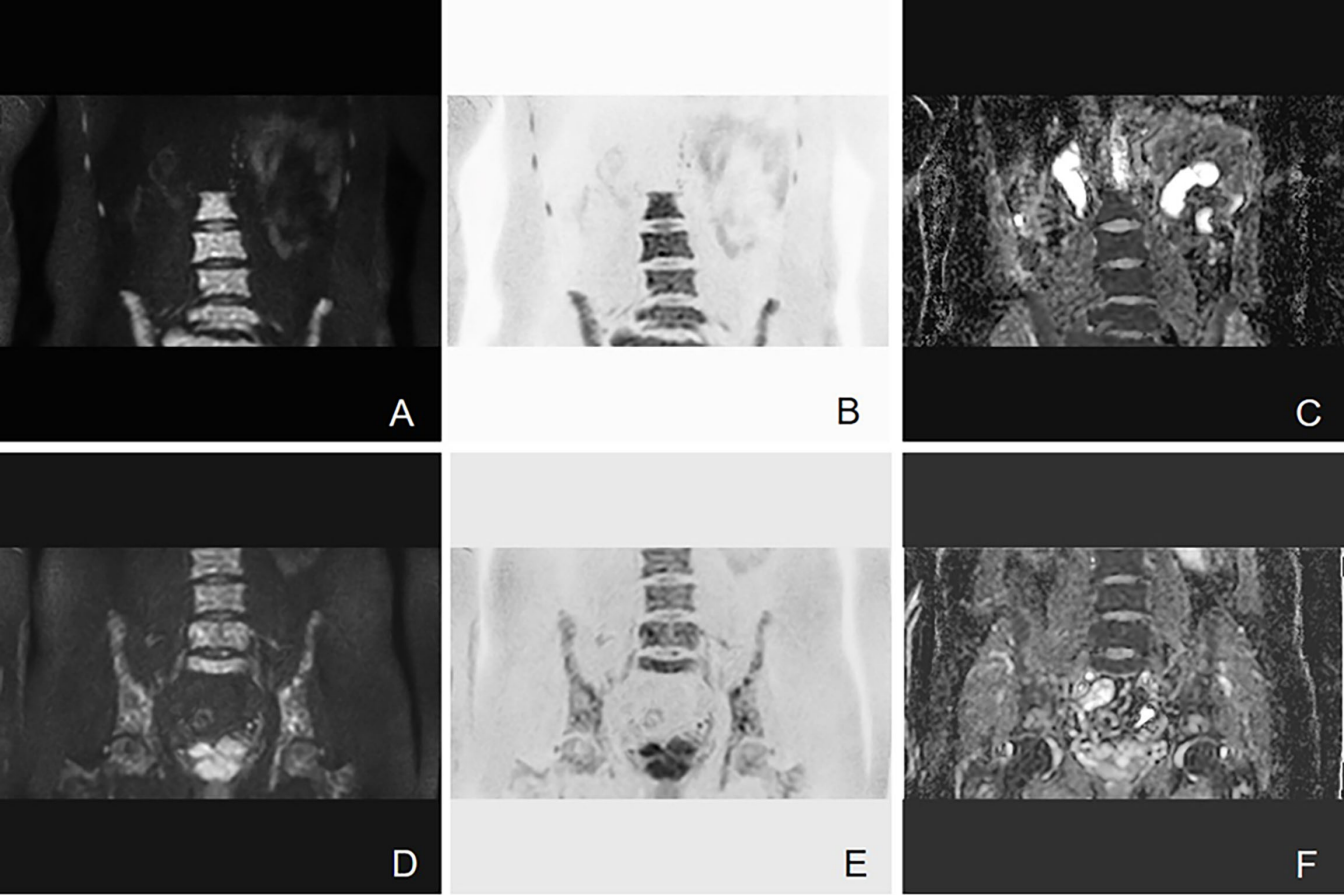
Example of DW-MRI image with treatment response of SD. A case of a 52-year-old man who was diagnosed with MM (IgA kappa light chain, RISS Stage III) is presented here. **(A–C)** Coronal images of DWI, inverted images, and ADC map at baseline visit. **(D–F)** Corresponding images of the same patient after four courses of induction chemotherapy (three courses of bortezomib, cyclophosphamide, and dexamethasone + one course of bortezomib, ralidomide, and dexamethasone). The efficacy of induction chemotherapy was evaluated as SD. Diffuse abnormal signal can be seen in the lumbar spine and pelvis. It has been shown that the high signal in the bone marrow of DWI sequence slightly decreased after induction chemotherapy. The mean ADC value of lesions in the scan range also increased from 0.693 × 10^−3^ to 0.775 × 10^−3^ mm^2^/s.

**Figure 5:**
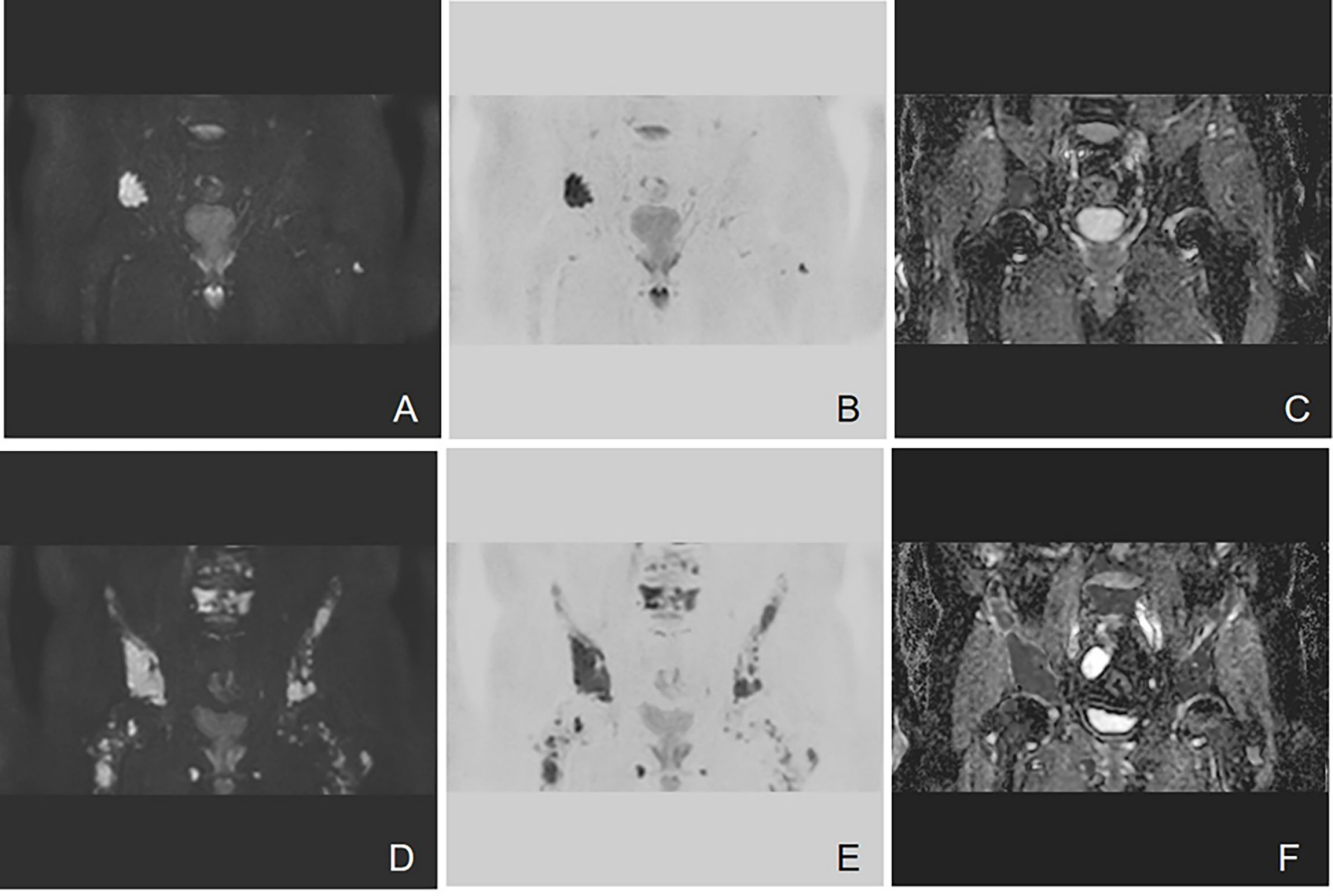
Example of DW-MRI image with treatment response of PD. A case of a 60-year-old man who was diagnosed with MM (type of IgG λ, RISS Stage II) is presented here. **(A–C)** Coronal images of DWI, inverted images, and ADC map at baseline visit. **(D–F)** Corresponding images of the same patient after three courses of induction chemotherapy (3 VCD). The treatment effect was evaluated as PD. Diffuse abnormal signal can be seen in the pelvis. It can be observed that the number of lesions increases and the scopes of most lesions expand. The mean ADC value of lesions in the scan range also increased from 0.743 × 10^−3^ to 0.881 × 10^−3^ mm^2^/s.

**Figure 6:**
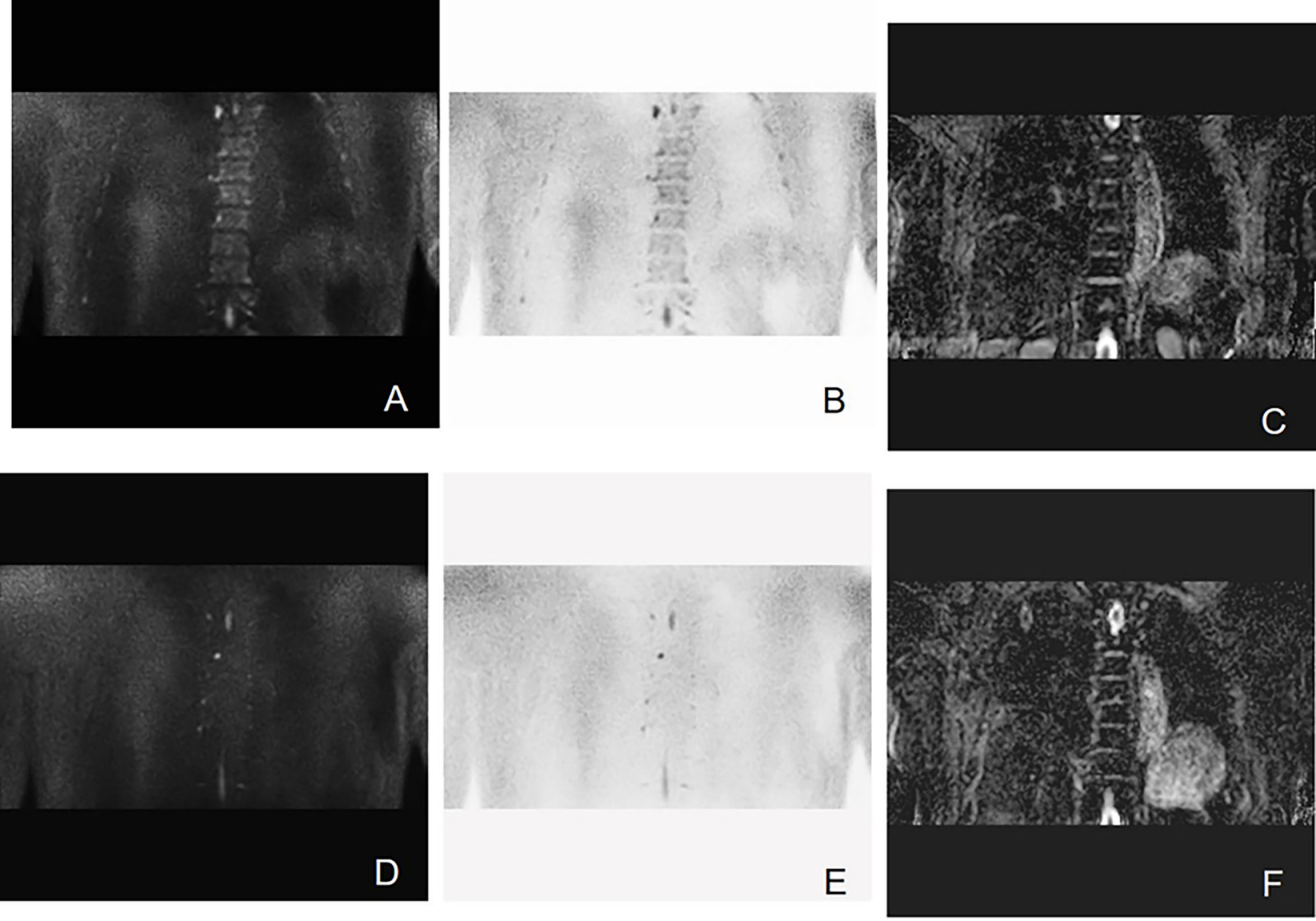
Example of DW-MRI image with treatment response of sCR. A case of 60-year-old man who was diagnosed with MM (type of IgG kappa, RISS Stage II) is inserted here. **(A–C)** Coronal images of DWI, inverted images, and ADC map at baseline visit. **(D–F)** Corresponding images of the same patient after three courses of induction chemotherapy (3 VCD + 1 VD). The treatment effect was evaluated as sCR. It has been observed that multiple focal abnormal signals can be seen on the baseline images. However, the number of lesions shown on the images after induction chemotherapy decreased significantly. The mean ADC value of lesions in the scan range also increased slightly from 0.462 × 10^−3^ to 0.570 × 10^−3^ mm^2^/s.

Bone involvement is one of the most prominent features of MM (18). As the information about cell metabolism allows the detection of active myeloma lesions, DW-MRI was found to have the highest sensitivity to bone marrow involvement (**Figure 3**). DW-MRI plays a more and more important role in the diagnosis, initial staging, and follow-up of patients with MM (**Figures 4**–**6**). At present, diffusion-weighted imaging is recommended for MM bone disease screening for clinical practice (11). The ADC measurement of DWI single index model is affected by many factors, such as blood perfusion, T2 penetration effect, and so on. Considering these factors, the motion of water molecules cannot be measured by a single exponential model. The motion of water molecules in tissue is not a simple Gaussian motion, and the measured ADC value is not consistent with the diffusion information on water molecules in tissue. The authenticity of the measured ADC value is related to the selection of *b*-value. The DWI sequence of larger *b*-value is more preferred to reflect real-water molecule movement. However, when the *b*-value is too large, the image’s signal-to-noise ratio will be relatively reduced. Taking into account the image quality and the authenticity of the ADC value, we use multi-*b*-value DWI imaging and double exponential model algorithm, which can well distinguish the diffusion movement of water molecules inside and outside the cells and the components of blood perfusion in tumor tissue (19).

In the univariate analysis, it can be seen that PFS and OS in the elder group (≥65 years) were shorter than those in the younger group (<65 years), despite confounding factors. However, in the multivariate analysis, age was not a statistically significant factor affecting PFS and OS. With the advancement of treatment method and new drugs in recent years, the prognosis of elderly MM patients has improved. For elderly patients, pretreatment with anti-CD38 antibody reasonable autologous stem cell transplantation is expected to achieve satisfactory treatment results (20). Although new agents are effective in prolonging survival in elderly patients with MM, elderly patients are more likely to have underlying diseases and develop treatment-related adverse events (21). Even though slightly different from previous studies, it is still an indisputable fact that elderly and frail myeloma patients may have shorter OS (22).

The mortality of patients with light chain MM exhibited significantly higher and the level of serum albumin was lower than other types (23). Similarly, it is believed that OS and the level of β2-MG were negatively correlated (*r* = −0.511, *p* = 0.01) (24). Our results are consistent with these previous studies; in our results, albumin and β2-MG levels were independent factors affecting PFS and OS. Different from our results, Okello et al. (6) found that higher LDH level (>225 U/L) was associated with poor survival rate of MM patients (HR = 3.3, 95% CI, 0.57–5.92; *p* = 0.029). MM with cytogenetic abnormalities is prone to recurrence and poor prognosis. Apoptosis escape and antitumor drug resistance in MM can be driven by genetic abnormalities, including P53 deletion, 1q21 amplification, and IGH rearrangement. It has been shown that IGH rearrangement is associated with significantly shortened OS, such as extramedullary lesions of MM, which means that OS is shorter than 6 months (25). The absence of IGH variable region was found to be associated with a significant reduction in 2-year PFS (*p* = 0.008) and adverse reactions to first-line treatment (*p* = 0.037) (26). It is believed that IGH rearrangement may affect the prognosis of MM not straightforwardly; however, to some extent, the disease progression is achieved by affecting the sensitivity of drug treatment (21, 22). For high-risk MM with p53 gene mutation, some new drugs can improve the prognosis of these relapsed MM by inhibiting p53 pathway (27, 28). In the continuous research progress, the survival status of MM patients with different types of gene variants is expected to be improved.

ADC value can provide DW-MRI with quantitative markers for tumor load, which is very important to evaluate the biological behavior of MM (29). The ADC value of DW-MRI is believed to explicitly reflect the cell density of MM lesions (10). The ADC value of MM bone lesions before initial treatment also reflects the tumor cell load at baseline. The yellow bone marrow in the patients with advanced MM decreases, proliferates actively, and increases abnormal plasma cells (30). These characteristics accelerate the diffusion of extracellular water molecules. Thus, MM bone lesions showed higher ADC values. DW-MRI and its derived ADC value can better evaluate the range of MM bone disease and reveal the condition of newly diagnosed MM patients. Although the currently recognized MM staging guidelines use only serological parameters, ADC value is a useful supplement for nonsecretory MM (31). In the case of false-positive β 2-MG, it can also help physicians more accurately understand the process of the disease. ADC value can reflect the curative effect (32). If the treatment plan can be adjusted to this, it is hoped that the prognosis can be improved.

It has been shown that lower ADC before treatment may be associated with better treatment response (13). This theory has may become one of the reasons why MM patients with lower ADC value obtained longer PFS and OS. Not only that, Sun et al. (15) have found that ADC value could increase with the progress of MM. The ADC value of MM bone lesions with RISS stage III was significantly higher than that of stage I and stage II (0.69 ± 0.22 vs. 0.44 ± 0.14, 0.69 ± 0.22 vs. 0.53 ± 0.21; ×10^−3^ mm^2^/s; all *p* < 0.05). The internal relationship between ADC value and MM stage may also be one of the internal reasons why high ADC value leading to short survival time. However, Dong et al. (33) have found that low ADC on DW-MRI maybe positively correlated with deep response in MM patients, but only in those without anemia. As for the reasons for the slight difference from our study, the influence of red bone marrow hyperplasia cannot be ruled out. The factors affecting the survival of MM may be a dynamic and far more sophisticated concept. The reality is that we may ignore the impact of clonal structure, epigenetics, immune microenvironment, and many other features on prognosis, which we do not consider due to lack of sufficient knowledge.

It has to be mentioned that this study contains some limitations. Firstly, the factors affecting the survival of MM included in the study are relatively limited. No single factor can evaluate survival alone. The inclusion of ADC value is a meaningful attempt, but more comprehensive factors need to be considered. Furthermore, this study was not stratified by different infiltration patterns of imaging, because the sample size of some classifications after stratification is small, which may lead to a deviation from the results. Finally, imaging omics is expected to be applied to research in the future, which will help to improve the effectiveness of the model for evaluating survival time.

Our study is the first to analyze PFS and OS in MM patients with different ADC values. The results show that DW-MRI may provide a reference for the survival time of MM patients. Univariate and multivariate analyses confirmed that ADC value could be independent prognostic predictors affecting PFS and OS in newly diagnosed MM patients. The ADC value as a predictive factor of the survival in MM would allow a multidimensional diagnosis, leading to a more personalized management and long-term treatment. Quantitative imaging index ADC value predicts the prognosis of MM, so as to create a new reliable standard for disease prediction and monitoring, which is an opportunity for the further research of MM imaging.

## Data Availability Statement

The datasets presented in this study can be found in online repositories. The names of the repository/repositories and accession number(s) can be found in the article/**Supplementary Material**.

## Ethics Statement

The studies involving human participants were reviewed and approved by the ethics committee of the First Hospital of Jilin University. The patients/participants provided their written informed consent to participate in this study.

## Author Contributions

BZ wrote the paper. BB reviewed the paper. YZ, LZ, and RZ established and coordinated the collection of all the data. JW has the primary responsibility for the final content. The authors read and approved the final manuscript.

## Funding

This work was supported by the Jilin Province Health Talents Foundation, China (No. JLSWSRCZX2020-047).

## Conflict of Interest

The authors declare that the research was conducted in the absence of any commercial or financial relationships that could be construed as a potential conflict of interest.

## Publisher’s Note

All claims expressed in this article are solely those of the authors and do not necessarily represent those of their affiliated organizations, or those of the publisher, the editors and the reviewers. Any product that may be evaluated in this article, or claim that may be made by its manufacturer, is not guaranteed or endorsed by the publisher.

## Abbreviations

ADC, apparent diffusion coefficient; DW-MRI, diffusion-weighted whole-body MRI; IMWG, International Myeloma Working Group; MM, multiple myeloma; OS, overall survival; PD, progressive disease; PFS, progression-free survival; sCR, strict complete response; SD, stable disease; VCD, bortezomib, cyclophosphamide, and dexamethasone; VD, bortezomib and dexamethasone; VRD, bortezomib, ralidomide, and dexamethasone; WB-DWI, whole-body diffusion-weighted imaging.
